# Phase-Sensitive Gaze Allocation in a Progressive Calligraphy Task

**DOI:** 10.3390/jemr19040069

**Published:** 2026-06-30

**Authors:** Yujun Liu, Nina Xie, Xutang Tong, Yuanyuan Wang

**Affiliations:** 1 Faculty of Business, Lingnan University, 8 Castle Peak Road, Tuen Mun, Hong Kong; yujunliu@ln.edu.hk (Y.L.); xutangtong@ln.hk (X.T.); 2 Department of Management, Faculty of Business, Lingnan University, 8 Castle Peak Road, Tuen Mun, Hong Kong; 3 Management and Strategy, Lee Shau Kee School of Business and Administration, Hong Kong Metropolitan University, Ho Man Tin, Kowloon, Hong Kong; cyywang@hkmu.edu.hk

**Keywords:** eye tracking, gaze allocation, Chinese calligraphy, external visual information, eye–hand coordination, response processes

## Abstract

Eye-movement studies of manual production often average gaze across an entire trial, obscuring how visual information use changes once actions begin. We separated the pre-writing and writing phases in a fixed progressive Chinese calligraphy task. Thirty-seven postgraduate students completed two style-guided transfer (SGT) pages, a worked example, and two evolution-based mapping (EBM) pages; 34 contributed usable gaze data. On SGT pages, reference allocation fell from 0.626 before writing to 0.131 during writing, whereas the share of reference viewing directed to diagnostic tokens rose from 0.473 to 0.601. On EBM pages, allocation to the cue-plus-context display fell from 0.825 to 0.447 after pen onset but remained substantial; cue share and context coverage also declined. Participant-level process blocks did not improve quality models. In exploratory page-level EBM analyses, greater pre-writing context coverage was associated with higher product quality. These findings identify pen onset as a useful boundary for analyzing visual information use in constrained production: external sampling is greatest before writing, and task-specific re-access persists during execution. Because the task order was fixed, page-family differences cannot be separated from practice or scaffolding. Phase-specific area-of-interest measures can therefore add process information to product scores without treating gaze as a direct measure of cognition.

## 1. Introduction

Design performance depends on how people inspect external representations, select relevant cues, maintain spatial relations, and monitor emerging outputs [1,2,3,4,5,6,7,8]. Analogical reasoning, visuospatial processing, attention, and working memory may support these operations [9,10,11,12,13,14,15,16,17,18,19], but product scores alone do not show how they are coordinated during performance.

Similar products may result from different patterns of information use. Validity and evidence-centered assessment frameworks therefore treat response processes as evidence for interpreting performance scores [20,21,22,23,24,25]. In externally guided production, comparable scores can be accompanied by different patterns of early visual sampling and later checking.

External representations are integral to design activity. Sketches, exemplars, and intermediate marks support comparison, reduce memory demands, and expose relations that can guide subsequent action [6,26,27,28,29,30,31,32]. Product ratings and retrospective reports capture outcomes and reflections but not the temporal organization of visual information use [2,5,27,33].

Eye tracking provides time-resolved evidence about where and when visual information is sampled [34,35,36]. Such evidence is interpretable only when AOIs, event-detection procedures, and task phases are defined in relation to the research question [37,38,39,40,41,42]. We therefore treat gaze as a behavioral record of visual information use, not as a direct measure of cognition.

Chinese character tasks are suitable for this analysis because visual form and spatial structure influence eye movements [43]. Zang et al. [44] showed that interword spacing affects landing positions in Chinese reading, while Bai et al. [45] showed that learned feature and location values can guide attentional capture. These findings motivate examination of how layout and cue value shape gaze allocation, but they do not determine the cognitive meaning of an individual fixation.

Eye-tracking studies of calligraphy, ideation, drawing, logo perception, and sketch mapping have also examined visual search and eye–hand coordination in production-oriented tasks [46,47,48,49,50]. Together, these studies support phase-specific analyses based on explicitly defined task regions and actions.

We examined gaze in a fixed calligraphy sequence containing SGT and EBM pages. On SGT pages, participants viewed five reference tokens and wrote a new character in the writing frame. On EBM pages, they viewed a highlighted Xiaozhuan form and its historical context before producing the target character. Every participant completed the same sequence and the same Hua worked example; page-family differences may therefore reflect task content, order, practice, or scaffolding. Primary analyses examined gaze allocation before and during writing. Secondary analyses examined cross-page associations and product quality. We tested whether the task provides phase-specific evidence about external information use rather than a general measure of design capacity.

## 2. Materials and Methods

### 2.1. Research Design

We used a fixed-sequence, within-participant design built around a progressive calligraphy task. We compared gaze allocation before and during writing as participants progressed through the fixed sequence. Gaze was analyzed separately before and during writing. Because all participants completed the same scaffolded order, page-family contrasts describe differences within that sequence and do not isolate task type from order, practice, or the worked example. Multivariable quality models were treated as secondary analyses.

### 2.2. Participants

Participant characteristics and exclusions are summarized in Table 1. Thirty-seven postgraduate students completed the task. Their mean age was 25.6 years (SD = 2.5; range = 23–33); 30 identified as female and seven as male. Participants completed the task using normal vision or their habitual visual correction. Two participants were excluded because their session-level tracking ratio was below 60%, leaving 35 participants with matched questionnaire, product-score, and AOI records. One further participant had no usable Jiang or Xiang page-phase records after segmentation and AOI pooling. The primary gaze-analysis sample therefore comprised 34 participants.

### 2.3. Stimuli and Task Sequence

Participants completed the fixed sequence: Baseline → Jiang (江) → Xiang (香) → Hua (画; EBM Worked Example) → Ma (马) → Que (雀). Jiang and Xiang constituted the SGT pages. On these pages, participants viewed a poem-strip reference containing five exemplar tokens and then wrote the target character in a dedicated grid. Hua served as an embedded worked example that introduced the logic of the later mapping task. Ma and Que constituted the EBM pages. On Ma and Que, participants used a highlighted Xiaozhuan cue and surrounding historical forms to infer and write the target character.

**Figure 1:**
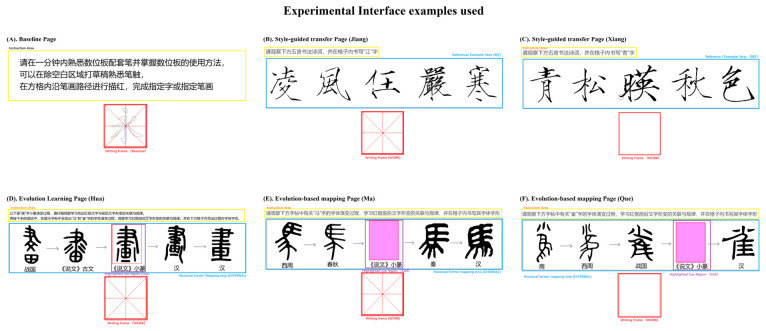
Experimental interface examples and AOI families. (**A**) shows the baseline page with the target character Yong (永) in Kaiti, or regular script; (**B**,**C**) show the SGT pages, which used Shoujin-style reference characters for style-guided transfer; (**D**) shows the worked-example page with Xiaozhuan; (**E**,**F**) show the EBM pages which used historical character forms for evolution-based mapping. The overlays indicate the main AOI families: reference or external display, highlighted cue, and writing frame. The Chinese character forms shown in the interfaces were task-relevant visual stimuli, and the SGT and EBM pages intentionally used different character-form styles to support different task demands.

Figure 1 shows the page layouts and the functional regions used in the gaze analyses. Because the task order was intentionally scaffolded rather than counterbalanced, contrasts between SGT and EBM pages should be interpreted as differences within this ordered sequence, not as pure task-family main effects.

### 2.4. Apparatus and Eye-Tracking Measures

Eye movements were recorded with a Tobii Pro Nano eye tracker (Tobii AB, Danderyd, Sweden) at 60 Hz. Stimuli were presented at 1920 × 1080 pixels on a 15.6-inch VivoBook Pro 15 laptop (ASUSTeK Computer Inc., Taipei, Taiwan). Written responses were recorded on a Wacom Intuos CTL-4100 tablet (Wacom Co., Ltd., Kazo-shi, Saitama, Japan) with an active area of 152 × 95 mm. Participants viewed the screen from approximately 50 cm and practiced writing on the tablet for about 1 min before the task.

Nine-point calibration and validation were completed at the start of each session. Calibration was repeated when validation error exceeded the pre-specified threshold of 2° or when validation quality was visually judged to be unreliable. Fixations were identified in Tobii Pro Lab 1.23 using the default Tobii I-VT (Fixation) filter for screen-based projects, with gap fill-in disabled, average eye selection, moving-median noise reduction, a 30°/s velocity threshold, adjacent-fixation merging within 75 ms and 0.5°, and a minimum fixation duration of 60 ms. Raw gaze samples were exported and archived for data retention purposes. The analyses reported here did not reclassify the raw sample stream or apply custom fixation, saccade, or scanpath algorithms. AOI summaries were exported for each participant, page, and phase. The reported analyses used only AOI-level fixation and visit summaries exported from Tobii Pro Lab 1.23; the archived raw samples were not reclassified.

### 2.5. Background Measures and Product Scoring

Participants completed a six-item non-verbal indicator battery; these scores were not treated as psychometric measures of design capacity. The battery included two reasoning measures (Picture Analogies and Grouping), three spatial-processing measures (Mirror Images, Dot Situation, and Shape Construction), and one attention/perceptual-organization measure (Embedded Images). An overall accuracy score was also retained.

Calligraphy knowledge was indexed by the means of five self-report items, and undergraduate background was coded as design/arts-related or non-design/arts-related.

Product quality was scored on structural correctness and layout/proportion. Structural correctness assessed recovery of the target structure and critical component relations. Layout/proportion assessed balance, spacing, and the distribution of the character within the writing frame. Each dimension was scored from 1 to 5. The rubric summary is reported in Table 2.

All focal pages were scored independently by three raters. All raters had more than eight years of experience studying calligraphy; two held Level-10 calligraphy certificates issued by the Art Development Center of the Ministry of Culture and Tourism, China, and one had more than five years of calligraphy teaching experience. Before scoring, the raters received the same written rubric and a standardized explanation of its anchors. Handwriting products were randomly ordered and anonymized. The raters scored them independently and did not discuss their ratings during scoring. The outcome used in subsequent analyses was the mean of the three raters’ page totals.

### 2.6. Procedure and Phase Segmentation

**Figure 2:**
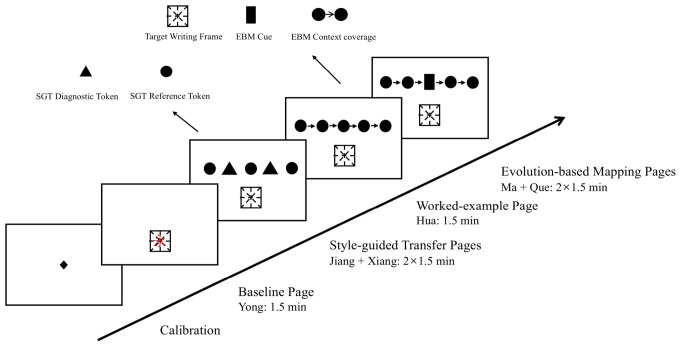
Experimental procedure and task-relevant display tokens. The figure shows the fixed task sequence and the task-relevant display elements used in the AOI-based analyses. Participants completed calibration, a 1.5 min baseline page, two 1.5 min style-guided transfer pages, a 1.5 min worked-example page, and two 1.5 min evolution-based mapping pages. The character Yong (永) denotes the baseline tracing character used for tablet familiarization. Circles denote SGT reference tokens, triangles denote SGT diagnostic tokens, the square/filled block denotes the EBM cue, linked circles denote EBM context coverage, and the grid square denotes the target writing frame.

**Figure 3:**
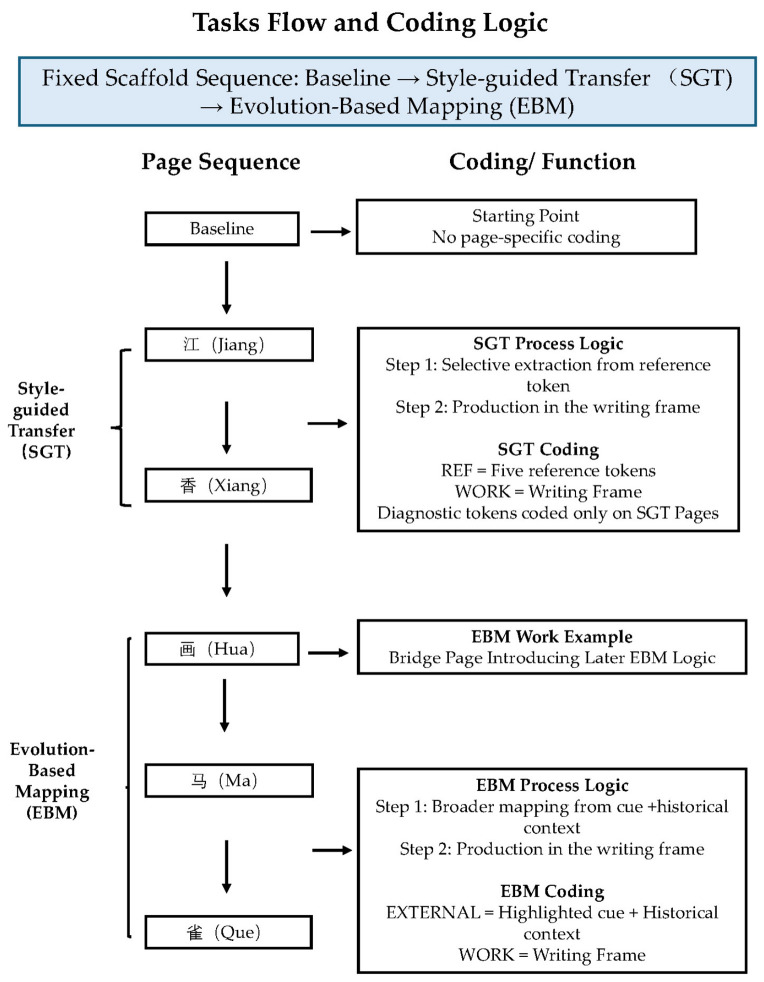
Task flow, scaffolded sequence, and coding logic. The figure summarizes the fixed Baseline-SGT-Hua-EBM sequence and the functional AOI families used for SGT and EBM pages.

Participants completed the fixed scaffolded sequence Baseline → Jiang → Xiang → Hua → Ma → Que (Figure 2 and Figure 3). The semi-fixed task sequence and the task-relevant display-token coding used for AOI pooling are illustrated in Figure 2. Jiang and Xiang were SGT pages, on which participants inspected poem-strip exemplars and then wrote the target character in a grid. Hua served as a worked example that introduced the logic of the later EBM pages. On Ma and Que, participants inspected a highlighted Xiaozhuan cue and the surrounding historical-form display before and during production of the target character. Participants were instructed to inspect the information available on each page and produce the target character in the writing frame.

The fixed order was retained to preserve the intended progression from transfer-based copying to history-based mapping. Accordingly, contrasts between SGT and EBM pages are interpreted within this ordered sequence. For each focal page, page onset was defined as the appearance of the stimulus screen, and pen onset was defined as the first recorded pen contact on the digital tablet. The pre-writing phase extended from page onset to pen onset and included all gazes recorded before the first stroke. The writing phase extended from pen onset to page completion and included writing and any subsequent looks to external regions.

### 2.7. Data Processing and Analysis

**Figure 4:**
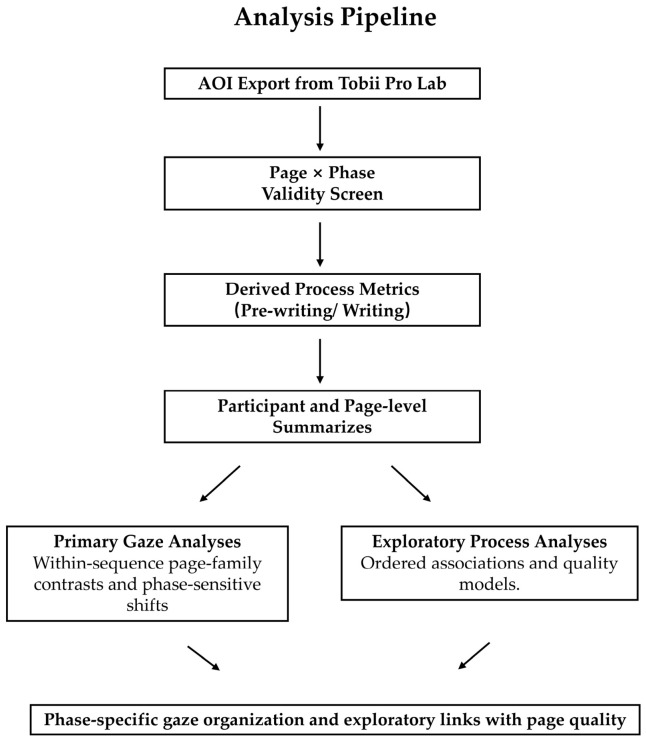
Analysis pipeline. AOI exports were screened at the page-phase level, converted into pre-writing and writing process metrics, and summarized at the participant and page levels for primary and secondary analyses.

The analysis pipeline is summarized in Figure 4. AOI exports were organized as participant-by-page-by-phase records. For each record, total fixation duration was pooled within functional AOI families. On SGT pages, REF comprised the five poem-strip reference tokens and WORK denoted the writing frame. On EBM pages, EXTERNAL was an analytic cue-plus-context family comprising the highlighted Xiaozhuan cue plus the surrounding historical-form context, and WORK again denoted the writing frame. The cue and context AOIs were mutually exclusive, so each fixation contributed once to EXTERNAL; instruction and off-task AOIs were excluded from the denominator.

A page-phase record was valid when the page-screen AOI contained fixation data and the relevant allocation denominator was greater than zero: REF + WORK for SGT and EXTERNAL + WORK for EBM. All allocation metrics were calculated from fixation duration within task-relevant AOI families. For SGT pages, REF allocation was calculated as $D_{REF}/(D_{REF}+D_{WORK})$, and WORK allocation as $D_{WORK}/(D_{REF}+D_{WORK})$. Diagnostic-token share was calculated as the proportion of reference-line fixation duration directed to the two a priori diagnostic tokens on each SGT page. For EBM pages, EXTERNAL allocation was calculated as $D_{EXTERNAL}/(D_{EXTERNAL}+D_{WORK})$, where $D_{EXTERNAL}$ was the summed fixation duration on the highlighted Xiaozhuan cue and the surrounding historical-context AOIs. Cue share was calculated as the proportion of external-display fixation duration allocated to the highlighted cue, and context coverage was calculated as the proportion of historical-context AOIs fixated at least once. The metrics describe task-specific visual information use and are not direct measures of cognition. Table 3 defines the derived metrics.

Participant-level summaries were the means of valid page-level metrics within each page family and phase. Primary hypotheses were tested with one-sample or paired-samples t tests. We report mean differences, 95% confidence intervals, Cohen’s dz, two-sided *p* values, and Benjamini–Hochberg adjusted q values. Because complementary allocation proportions contain identical information, only one measure from each complementary pair was tested inferentially: REF allocation for SGT pages and EXTERNAL allocation for EBM pages.

Secondary analyses examined cross-page associations and product quality. Page-level models used Gaussian-identity GEE with participant as the clustering unit, an exchangeable working correlation, and robust sandwich standard errors. Continuous predictors were z-standardized. Missing page records were analyzed by available-case analysis; no values were imputed. Statistical analyses were conducted in Python 3.12.13 using statsmodels 0.14.6; Tobii Pro Lab version 1.23 was used for AOI definition and export of fixation and visit summaries.

## 3. Results

### 3.1. Data Retention, Valid Records, and Scoring Reliability

Thirty-four participants contributed to the primary gaze analyses. Valid page records varied by page and phase after the application of the page-phase criteria; Table 4 reports the corresponding sample sizes.

Inter-rater reliability was estimated with a two-way random-effects, absolute-agreement model. Across 140 participant-page records, ICC(2,1) was 0.690 and ICC(2,3) was 0.870. Page-specific average-measure ICCs were 0.889 for Jiang, 0.925 for Xiang, 0.725 for Ma, and 0.845 for Que (Table 5).

### 3.2. Primary Gaze Analyses: Phase Shifts and Pre-Writing Evidence Organization

**Figure 5:**
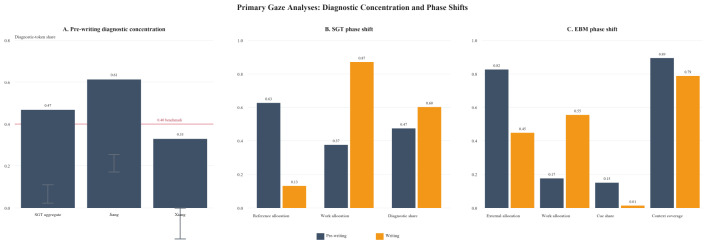
Primary gaze analyses. (**A**) shows pre-writing diagnostic-token concentration against the 0.40 equal-share benchmark. (**B**,**C**) show the phase shifts from pre-writing to writing for the SGT and EBM page families.

Before writing, the aggregate SGT diagnostic-token share was 0.466, exceeding the 0.40 equal-token benchmark, t(33) = 3.089, *p* = 0.004, q = 0.005, d<sub>z</sub> = 0.530 (Figure 5A). The pattern differed by prompt: Jiang averaged 0.613, whereas Xiang averaged 0.328. EBM external allocation exceeded SGT reference allocation (0.825 vs. 0.626), t(33) = 6.747, *p* < 0.001, q < 0.001, d<sub>z</sub> = 1.157. This cross-family comparison is functional and does not control for AOI area or visual content.

On SGT pages, REF allocation decreased from 0.626 before writing to 0.131 during writing, t(33) = 19.287, *p* < 0.001, q < 0.001, d<sub>z</sub> = 3.308 (Figure 5B). Diagnostic-token share increased from 0.473 to 0.601, t(29) = −2.172, *p* = 0.038, q = 0.043, d<sub>z</sub> = −0.396. Thus, less gaze was directed to the reference strip during writing, but more of that viewing fell on the diagnostic tokens.

On EBM pages, EXTERNAL allocation decreased from 0.825 before writing to 0.447 during writing, t(33) = 16.159, *p* < 0.001, q < 0.001, d<sub>z</sub> = 2.771 (Figure 5C). Cue share decreased from 0.150 to 0.013, t(33) = 9.822, *p* < 0.001, q < 0.001, d<sub>z</sub> = 1.684, and context coverage decreased from 0.893 to 0.787, t(33) = 3.189, *p* = 0.003, q = 0.005, d<sub>z</sub> = 0.547. External viewing, therefore, declined after pen onset but remained substantial during production.

Across both page families, pen onset was followed by a marked shift from external regions to the writing frame. Diagnostic-token concentration was driven mainly by Jiang, whereas EBM pages showed greater functional allocation to the cue-plus-context region before writing. All nine unique primary contrasts remained significant after Benjamini–Hochberg adjustment (largest q = 0.048; Table 6).

### 3.3. Exploratory Process Findings

Exploratory analyses tested whether SGT pre-writing reference allocation was associated with later EBM gaze. Higher SGT reference allocation was associated with higher EBM EXTERNAL allocation (B = 0.036, *p* = 0.013) and lower cue share (B = −0.026, *p* = 0.027). Because WORK is the mathematical complement of EXTERNAL, the duplicate WORK model was omitted. These within-sequence associations do not establish transfer or causation; full models are reported in Appendix A.

**Figure 6:**
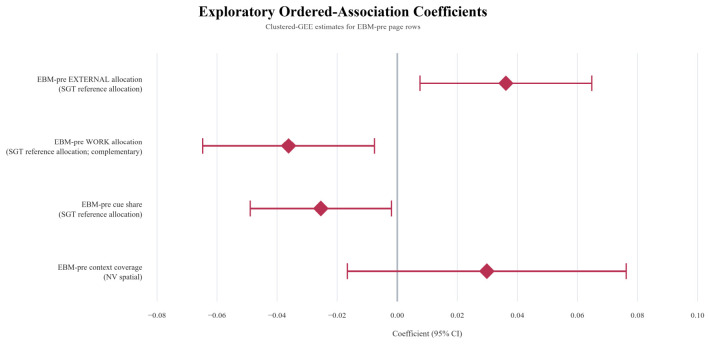
Diamonds show focal GEE coefficients with 95% confidence intervals for EBM pre-writing page records. Estimates describe within-sequence associations, not transfer or causal effects.

Figure 6 visualizes selected coefficients from the secondary ordered-association models. Because all participants completed the same fixed scaffolded sequence, these estimates describe within-sequence associations.

Participant-level process blocks did not increase the explained variance for Style quality (ΔR^2^ = 0.011, *p* = 0.966) or Evolution quality (ΔR^2^ = 0.144, *p* = 0.193). In the exploratory page-level EBM model, NV attention (B = 0.186, *p* = 0.017), cue share (B = 0.272, *p* = 0.013), and context coverage (B = 0.271, *p* < 0.001) were positively associated with quality; SGT reference allocation was not (*p* = 0.215; Appendix A).

## 4. Discussion

Pen onset marked a clear change in gaze allocation. On SGT pages, reference viewing decreased during writing while the remaining reference viewing became more concentrated on diagnostic tokens. On EBM pages, cue-plus-context viewing also decreased but remained substantial during writing. Quality associations were confined to exploratory page-level EBM models; participant-level process blocks were not significant.

### 4.1. Phase-Related Shifts in Gaze Allocation

Across both page families, pen onset marked a shift from external information gathering to writing frame allocation. This repeated shift supports pen onset as a practical boundary between the pre-writing and writing phases.

The interfaces differed in the external information they presented. SGT pages contained five contemporary reference tokens, whereas EBM pages contained a highlighted cue and several historical forms. The observed allocation differences may therefore reflect page layout, informational content, sequence position, or the Hua scaffold. The present design cannot separate these influences.

Gaze allocation showed when and where participants sampled the display, information not contained in page-quality scores. The present measures do not, however, identify complete individual strategies.

### 4.2. Within-Class Heterogeneity and Page Identity

The aggregate SGT result masked a marked Jiang–Xiang difference: diagnostic-token concentration was evident for Jiang but not Xiang. Because prompt properties were not manipulated, the source of this difference remains unresolved. Future task development should validate visual complexity, AOI area, diagnostic-token utility, and page-level gaze patterns before combining prompts into a task-family score.

### 4.3. EBM Gaze Measures and Page Quality

Participant-level process blocks did not significantly improve the Style or Evolution quality models. In exploratory page-level EBM analyses, pre-writing context coverage, cue share, and the NV attention indicator were positively associated with page quality. These estimates are based on 65 page records from 34 participant clusters and require replication.

SGT pre-writing reference allocation was not associated with evolution-page quality in the expanded model. The observed quality associations were limited to concurrent EBM measures.

### 4.4. Implications for Process-Sensitive Assessment

The task provides a compact way to describe how gaze is divided between external information and ongoing production. The present evidence does not support ranking or individual diagnosis. Any formative use would require replication across prompts, samples, and counterbalanced sequences, together with evidence that gaze metrics add value beyond product scores.

## 5. Limitations and Future Work

The fixed sequence prevents separation of page-family effects from order, practice, adaptation, and the Hua scaffold. The primary sample included 34 participants, and the exploratory EBM models used 65 page records from 34 clusters. AOI summaries from a 60 Hz tracker did not support fine-grained temporal or saccadic analyses. SGT REF and EBM EXTERNAL also differed in area, content, and salience. Only two prompts represented each family, and Jiang and Xiang produced different patterns. Finally, agreement was lower for Ma than for the other pages, increasing uncertainty in its quality score.

Future studies should counterbalance task order, manipulate scaffold availability independently, and use larger, prevalidated prompt sets. Larger samples would permit participant and prompt effects to be modeled jointly. Future analyses of the archived raw gaze samples should report calibration records, fixation-filter settings, and synchronized pen events. Any educational application should be evaluated prospectively against product scoring.

## 6. Conclusions

In this fixed calligraphy sequence, pen onset marked a shift from external information gathering to writing-frame allocation. SGT and EBM pages showed different gaze patterns, but task content could not be separated from order or scaffolding. Exploratory page-level analyses linked EBM context coverage to quality, whereas participant-level process blocks were not significant. Phase-specific eye tracking therefore adds information about visual sampling during constrained writing, but it does not provide a general measure of design cognition or capacity. Given the modest sample, the multivariable findings should be treated as preliminary and require replication in larger, counterbalanced samples.

## Figures and Tables

**Table 1:** Participant demographics and available background characteristics.

Characteristic	Statistic
Age, years: mean (SD), range	25.6 (2.5), 23–33
Gender: female/male	30/7
Self-reported vision problem: yes/no/missing	15/21/1
Habitual correction use: glasses/contact lenses/lens implant/none/missing	16/10/1/9/1
Major background: design-or-arts-related/non-design-or-arts related	23/14
Calligraphy Knowledge index (5-item, 1–5 for weak to strong): mean (SD), range	1.76 (0.55), 1.0–3.6
Calligraphy Skill level (5-item, 1–5 for weak to strong): mean (SD), range	1.59 (0.80), 1–4

**Table 2:** Summary of product-quality rubric.

Dimension	Anchored Description
Structural correctness	1 = severe structural mismatch; 5 = page-appropriate recovery of the target structure and relation-bearing components
Layout/proportion	1 = severe compression or imbalance; 5 = appropriate proportion, spacing, and page-specific expansion

**Table 3:** Mathematical definitions of derived eye tracking metrics.

Metric	Page Family	Definition	Interpretation
REF allocation	SGT	$\frac{D_{REF}}{\left(D_{REF}+D_{WORK}\right)}$	Proportion of task-relevant fixation duration allocated to the poem-strip reference tokens.
WORK allocation	SGT	$\frac{D_{WORK}}{\left(D_{REF}+D_{WORK}\right)}$	Proportion of task-relevant fixation duration allocated to the writing frame.
Diagnostic-token share	SGT	$\frac{D_{DIAG}}{D_{REF}}$	Selective reference viewing toward diagnostic tokens. The equal-share benchmark was 0.40.
EXTERNAL allocation	EBM	$\frac{D_{EXTERNAL}}{\left(D_{EXTERNAL}+D_{WORK}\right)}$	Proportion of task-relevant fixation duration allocated to the cue-plus-context display.
WORK allocation	EBM	$\frac{D_{WORK}}{\left(D_{EXTERNAL}+D_{WORK}\right)}$	Proportion of task-relevant fixation duration allocated to the writing frame.
Cue share	EBM	$\frac{D_{CUE}}{D_{EXTERNAL}}$	Concentration of external-display viewing on the highlighted Xiaozhuan cue.
Context coverage	EBM	$\frac{\sum_{K=1}^{K}I(D_{CONTEXT,K}>0)}{K},\;\;K=4$	Proportion of historical-context AOIs fixated at least once.

Note. D denotes total fixation duration within the specified AOI family. *D_EXTERNAL_* is the sum of fixation duration on the highlighted cue and historical-context AOIs. $D_{EXTERNAL}$ = $D_{CUE}+D_{CONTEXT}$. Ling (凌) and Ren (任) were designated as diagnostic tokens for Jiang (江); Qing (青) and Qiu (秋) were designated for Xiang (香). The 0.40 benchmark for SGT diagnostic-token share reflects two diagnostic tokens among five poem-strip reference tokens under equal token allocation.

**Table 4:** Analysis sample and valid page phase records after screening.

Data Layer	Retention Criterion	Available Records
Task completion	Completed the fixed Baseline-SGT-Hua-EBM sequence	37 participants
Eye-tracking quality screen	Tracking ratio ≥ 60% and matched AOI export	35 participants
Primary gaze-analysis sample	Usable SGT phase data after segmentation and AOI pooling	34 participants
Pre-writing page records	Valid screen AOI and task-specific denominator	Jiang = 32; Xiang = 33; Ma = 32; Que = 32
Writing page records	Valid screen AOI and task-specific denominator	Jiang = 31; Xiang = 33; Ma = 33; Que = 30
Product-score reliability	Three independent raters across four focal pages	140 participant-page records

**Table 5:** Three-rater agreement for rubric-based page quality totals.

Page	N	ICC(2,1) [95% CI]	ICC(2,3) [95% CI]	F(df1, df2)	*p*
Jiang	35	0.727 [0.579, 0.839]	0.889 [0.805, 0.940]	F(34, 68) = 9.426	<0.001
Xiang	35	0.803 [0.579, 0.839]	0.925 [0.868, 0.959]	F(34, 68) = 13.193	<0.001
Ma	35	0.468 [0.268, 0.656]	0.725 [0.523, 0.851]	F(34, 68) = 3.816	<0.001
Que	35	0.645 [0.473, 0.785]	0.845 [0.729, 0.916]	F(34, 68) = 6.707	<0.001
Overall page records	140	0.690 [0.611, 0.759]	0.870 [0.825, 0.904]	F(139, 278) = 8.075	<0.001

Note. ICCs were computed on page-quality totals after summing the two rubric dimensions for each rater. ICC(2,1) denotes the single-rater absolute-agreement reliability; ICC(2,3) denotes the reliability of the mean score across the three raters. Values in brackets are 95% confidence intervals. N refers to participant-page scoring records.

**Table 6:** Primary gaze contrasts for phase-sensitive shifts and pre-writing evidence organization.

Analysis	N	Mean ^a^	Mean ^b^/Benchmark	Mean Diff.	95% CI	t (df)	*p*	dz	FDR q
SGT REF pre-writing vs. writing	34	0.626	0.131	0.496	[0.444, 0.548]	19.287 (33)	<0.001	3.308	<0.001
SGT diagnostic share pre-writing vs. writing	30	0.473	0.601	−0.128	[−0.249, −0.007]	−2.172 (29)	0.038	−0.396	0.043
EBM EXTERNAL pre-writing vs. writing	34	0.825	0.447	0.378	[0.330, 0.425]	16.159 (33)	<0.001	2.771	<0.001
EBM cue share pre-writing vs. writing	34	0.150	0.013	0.136	[0.108, 0.165]	9.822 (33)	<0.001	1.684	<0.001
EBM context coverage pre-writing vs. writing	34	0.893	0.787	0.107	[0.039, 0.175]	3.189 (33)	0.003	0.547	0.005
SGT diagnostic share vs. 0.40 aggregate	34	0.466	0.400	0.066	[0.023, 0.110]	3.089 (33)	0.004	0.530	0.005
Jiang diagnostic share vs. 0.40	32	0.613	0.400	0.213	[0.170, 0.255]	10.211 (31)	<0.001	1.805	<0.001
Xiang diagnostic share vs. 0.40	33	0.328	0.400	−0.072	[−0.144, −0.001]	−2.056 (32)	0.048	−0.358	0.048
EBM external pre-writing vs. SGT REF pre-writing	34	0.825	0.626	0.198	[0.138, 0.258]	6.747 (33)	<0.001	1.157	<0.001

ᵃ First value in the contrast. ᵇ Second value in the contrast, or the benchmark value when a benchmark was tested. For phase-shift contrasts, the two means correspond to pre-writing and writing, respectively. For cross-family contrasts, the two means correspond to the measures named in the Analysis column.

## Data Availability

The data supporting this study are available from the corresponding author upon reasonable request. Raw eye-tracking and handwriting records may contain potentially identifiable behavioral traces and cannot be made publicly available under the approved consent and data-management arrangements. Cleaned analytic datasets, data dictionaries, AOI definitions, the scoring rubric, and analysis scripts are available upon reasonable request.

## Abbreviations

The following abbreviations are used in this manuscript:

AOI	Area of interest
CI	Confidence interval
EBM	Evolution-based mapping
EXTERNAL	Highlighted Xiaozhuan cue plus surrounding historical-form context
GEE	Generalized estimating equation
NV	Non-verbal
OLS	Ordinary least squares
REF	Reference tokens in the poem strip
SGT	Style-guided transfer
WORK	Writing frame

## Appendix A

The models reported in Appendix A were exploratory and were not used for primary inference. Continuous predictors were z-standardized before modeling. GEE models used participants as the clustering unit, a Gaussian identity link, an exchangeable working correlation, and robust sandwich standard errors. Model-specific available-case estimation was used.

**Table A1:** Exploratory ordered-association GEE models for EBM pre-writing page rows.

Outcome	Predictor	b	Robust SE	95% CI	*p*
EXTERNAL allocation	Intercept	0.815	0.020	[0.775, 0.854]	<0.001
EXTERNAL allocation	SGT REF allocation, pre-writing	0.036	0.015	[0.008, 0.065]	0.013
EXTERNAL allocation	Page: Que vs. Ma	0.022	0.022	[−0.022, 0.066]	0.326
Cue share	Intercept	0.218	0.023	[0.173, 0.263]	<0.001
Cue share	SGT REF allocation, pre-writing	−0.026	0.012	[−0.049, −0.003]	0.027
Cue share	Page: Que vs. Ma	−0.139	0.025	[−0.187, −0.090]	<0.001
Context coverage	Intercept	0.886	0.025	[0.837, 0.935]	<0.001
Context coverage	NV spatial indicator	0.029	0.023	[−0.016, 0.075]	0.208
Context coverage	Page: Que vs. Ma	0.020	0.039	[−0.056, 0.097]	0.605

**Table A2:** Participant-level exploratory quality block models.

Outcome	N	Background-Block (R^2^)	Full-Model (R^2^)	(\Delta R^2^)	(F_{\Delta})	*p*
Style quality	30	0.017	0.028	0.011	0.087	0.966
Evolution quality	34	0.090	0.234	0.144	1.687	0.193

**Table A3:** Exploratory page-level EBM quality GEE model.

Predictor	b	Robust SE	95% CI	*p*
Intercept	6.104	0.160	[5.789, 6.418]	<0.001
Page: Que vs. Ma	0.214	0.204	[−0.185, 0.614]	0.293
NV attention indicator	0.186	0.078	[0.033, 0.340]	0.017
SGT REF allocation, pre-writing	0.241	0.194	[−0.140, 0.622]	0.215
EBM cue share, pre-writing	0.272	0.110	[0.058, 0.487]	0.013
EBM context coverage, pre-writing	0.271	0.068	[0.137, 0.404]	<0.001
